# Wolf-Hirschhorn Syndrome: Clinical and Genetic Study of 7 New Cases, and Mini Review

**DOI:** 10.3390/children8090751

**Published:** 2021-08-30

**Authors:** Eva-Cristiana Gavril, Alina Costina Luca, Alexandrina-Stefania Curpan, Roxana Popescu, Irina Resmerita, Monica Cristina Panzaru, Lacramioara Ionela Butnariu, Eusebiu Vlad Gorduza, Mihaela Gramescu, Cristina Rusu

**Affiliations:** 1 Department of Medical Genetics, Faculty of Medicine, “Grigore T. Popa” University of Medicine and Pharmacy, University Street, No 16, 700115 Iasi, Romania; evagavril@yahoo.com (E.-C.G.); roxana.popescu2014@gmail.com (R.P.); irina.resmerita@umfiasi.ro (I.R.); monica.panzaru@umfiasi.ro (M.C.P.); lacrybutnariu@gmail.com (L.I.B.); vgord@mail.com (E.V.G.); mihaelagramescu@yahoo.com (M.G.); abcrusu@gmail.com (C.R.); 2 Department of Pediatric Cardiology, Faculty of Medicine, “Grigore T. Popa” University of Medicine and Pharmacy, University Street, No 16, 700115 Iasi, Romania; 3 “St. Mary” Children’s Hospital, Vasile Lupu Street, No 62-64, 700309 Iasi, Romania; 4 Department of Biology, Faculty of Biology, “Alexandru Ioan Cuza” University of Iasi, Bd. Carol I, 20A, 700505 Iasi, Romania

**Keywords:** Wolf–Hirschhorn Syndrome, 4p deletion, craniofacial dysmorphism, growth retardation, IUGR

## Abstract

Wolf–Hirschhorn syndrome (WHS), a rare disorder determined by distal 4p deletion, is characterized by a pre and postnatal growth retardation, hypotonia, intellectual disability, epilepsy, craniofacial dysmorphism, and congenital fusion anomalies. The clinical aspects are dependent on the deletion’ size. Our aim was to identify rare specific characteristics in a cohort of seven cases with 4p deletion and to assess the utility of Multiplex ligation-dependent probe amplification (MLPA) (cheap and sensitive test)—combined kits—as a diagnostic test and selection tool for cases that require other investigations (chromosomal microarray analysis—CMA, karyotype). For all cases we conducted a clinical examination with the main features identified: facial dysmorphism, intellectual disability, postnatal development delay, cardiac defects and hypotonia. In some cases, we observed seizures, structural brain abnormalities, immunodeficiencies, and renal anomalies. Prenatal growth retardation was detected in a relatively small number of cases, but postnatal growth failure was a constant feature. In all cases, the clinical diagnosis was confirmed by genetic analyses: karyotype and/or MLPA. In conclusion, renal and brain defects, as well as immunodeficiency are rare manifestations and should be looked for. Although CMA is the standard test, in our experience, MLPA is also a reliable screening method as the identified cases were either confirmed by MLPA or selected for further investigations.

## 1. Introduction

Wolf–Hirschhorn syndrome (WHS) is a rare contiguous gene deletion syndrome (prevalence of 1:20,000–50,000 births, with a female to male ratio of 2:1 induced by the absence of the distal portion of the short arm of chromosome 4 [1,2]. WHS presents a characteristic phenotype that includes intrauterine growth retardation and later on short stature, low weight, hypotonia, intellectual disability, epilepsy, and specific craniofacial dysmorphism (“Greek warrior helmet”). Other associated clinical manifestations include skeletal anomalies, congenital heart defects (septal defects and pulmonary stenosis), eye abnormalities, hearing loss, genitourinary tract defects, and immunological disorders [3]. The suggestive craniofacial features include wide nasal bridge continuing to the forehead, high anterior hairline with prominent glabella, highly arched eyebrows, widely spaced eyes, epicanthus, short philtrum, downturned corners of the month and micrognathia. Most of the patients have microcephaly and poorly formed ears (lobeless pinnae, underdeveloped or absent cartilage) as well as low-set or posteriorly angulated with pits/tags [3]. The genetic defect is usually a partial deletion of the distal short arm of chromosome 4, but WHS phenotype could be generated also by complex chromosomal rearrangements, like translocations or ring chromosomes. The unbalanced translocations can be *de novo* or inherited from a parent with a balanced rearrangement. The most frequently observed translocations are: (1) involving a rearrangement t(4p;8p), but t(4p;7p), t(4p;11p), t(4p;20q), t(4p;21q) and t(4p;12p, (2) inverted duplications associated with terminal deletions on the same 4p arm or 3, unbalanced pericentric inversions are cited [3,4]. There is a great variability in the extent of the deletion, ranging from less than 2 Megabases, (Mb) up to 30 Mb. There are three different categories of WHS phenotypes based on the 4p deletion size: a small deletion (not exceeding 3.5 Mb) which is usually associated with a mild phenotype (clinical manifestations are limited to the typical facial dysmorphism, growth delay, mild intellectual disability, and seizures). The large deletion (5 to 18 Mb) is the most frequent and it is associated with more severe manifestations including hypotonia, severe growth delay, significant neurodevelopmental impairment, and major malformations on top of the ones from the mild type, whereas the very large deletion (exceeding 22–25 Mb) is characterized by the most severe phenotype and is frequently hard to recognize as WHS [4]. The genomic defect and the pattern of seizures are the most important prognostic factors for the neurodevelopmental issues [4]. The WHS minimal critical region (MCR) is located approximately 2 Mb from the telomere 4p, within band 4p16.3 [5] (a region of 200–750 kilobases (kb) rich in genes—*WHSC1, LETM1, NSD2, CPLX1, PIGG* being usually deleted) [6,7]. The diagnosis requires clinical examination and genetic analysis; deletions greater than 5 MB can be identified by karyotype, but for smaller deletions (microdeletions) molecular diagnostic—CMA, MLPA, fluorescent in situ hybridization (FISH)— is necessary. Standard chromosomal analysis (routine and high resolution) is able to detect about 50–60% of the deletions, whereas FISH, using a *WHSCR* probe, detects more than 95%. Chromosomal microarray (CMA) detects all currently known deletions of the *WHSCR* and can determine if the deletion is pure or part of a more complex rearrangement. Standard cytogenetics may accompany the CMA to characterize any complex aberration if present. MLPA is a multiplex PCR assay that uses up to 40 probes, each specific for a different DNA sequence [8]. In recent years, MLPA has become a widely used technique in laboratories performing genetics testing for the molecular diagnosis of several diseases. MLPA is a high throughput analysis and allows a comprehensive identification of gene dosages with relatively low cost equipment and reagents [9]. A combined MLPA(combination of specific kits for microdeletion screening, followed up with specific kits for microdeletion confirmation) testing can be used to detect microdeletions that may not be identified by FISH. As there are no specific treatments available, supportive management can be useful considering WHS has a high mortality rate (30% within the first two years of life). The most common complications leading to death are respiratory tract infections and congenital heart diseases [1].

The aim of our study was to identify rare specific characteristics of WHS, to determine if there are major differences based on the size of the deletions and whether there is a correlation between deletion size and phenotypic severity, as well as trying to establish MLPA as a reliable tool for the diagnosis oh WHS.

## 2. Materials and Methods

### 2.1. Ethical Compliance

All subjects gave their informed consent for inclusion before they participated in the study. The study was conducted in accordance with the Declaration of Helsinki, and the protocol was approved by the Ethics Committee of “Grigore T. Popa” University of medicine and Pharmacy, Iasi (approval at 7 July 2020). All personal information of the patients has been anonymized, they have been given code numbers and releasing any personal details has been avoided unless relevant to the study at hand. An informed consent was obtained from patients (or relatives/legal guardians) before the beginning of the study. Participation in the current study was voluntary.

### 2.2. Patient Recruitment

We selected from our available medical files a cohort of seven unrelated patients with cytogenetic or molecular diagnosis of WHS confirmed by means of MLPA kits and confirmed by FISH where needed. This group was composed of four boys and three girls, with ages between 1 and 13 years. The cases were evaluated at “St. Mary” Children Hospital, Iasi, and Iasi Regional Medical Genetics Center, for an average period of 4 years.

### 2.3. Research Methodology

Patients underwent a complete history and physical examination. All patients were subjected to karyotype investigations (using peripheral blood) and MLPA (P096, P245 MLPA Kit for microdeletion screening, following-up with Kit P264, P358 for microdeletion confirmation)—MRC Holland^®^. Karyotype analysis was performed on peripheral blood lymphocyte cultures. At least 16 metaphases with 550 bands were evaluated under a microscope, and the captured images were analyzed using CytoVision. DNA extraction was performed from peripheral blood using the Genomic DNA Purification Wizard. The DNA was precipitated in 70% alcohol, resuspended in hydration solution, and stored at 4 °C. The standard MLPA analysis was performed according to the manufacturer’s recommendations: MRCHolland, Amsterdam, The Netherlands. Genomic DNA (100 ng) was denatured and hybridized with the probes in the kit, at 60 °C for 18–20 h. After a binding step at 54 °C for 15 min, PCR amplification was performed with 1 µL universal primers labeled with Cy5 in a SensoQuest thermocycler (The Kem-En-Tec Nordic, Denmark). The amplification was done after the following program: 35 cycles of denaturation (95 °C/30 s), hybridization (60 °C/30 s) and elongation (72 °C/60 s), completed with final elongation 72 °C/20 min. Fluorescent amplification products were subsequently separated by capillary electrophoresis in a CEQ sequencer 8000 GeXP Genetic Analysis System (Beckman Coulter). The number of copies of DNA was estimated using the Coffalyser.net program, which calculates the ratio of fluorescence intensity from patients to that of control cases for each target sequence. The results were expressed as the ratio of the number of copies of alleles to control patients (normal): the ratio obtained is 1 if both alleles are present, ~0.5–0.7 if an allele is absent and ~1.3–1.5 respectively if an allele is duplicated. A normal range was set at a threshold of 0.8 for deletion and 1.2 for duplication.

## 3. Results

### 3.1. Small Deletions (<3 Mb)

CASE 1

Case 1 is a 13-year-old boy, first child of a young, unrelated, apparently healthy couple, with no similar cases in the family. The pregnancy was uneventful, fetal ultrasound scan (36 weeks of amenorrhea (WA) revealed a corpus callosum agenesis. The birth was at full term, by cesarean section, (weight (Wt) = 3600 g, height (Ht) = 52 cm, occipital-frontal circumference (OFC) = 36.5 cm, Apgar score 8). Postnatal development was severely delayed (raised head at 18months (Mo), sat without support at 2 years (Y), no speech at age 13Y); the patient has recurrent respiratory infections. Physical evaluation at 13 years and 7 months (Wt: −0.89 standard deviation (SD), Ht: −2.39 SD, OFC: −1.23 SD) revealed: dysmorphic face, defect of lacrimal system, a small palate defect, pectus carinatum, diastasis recti, hypospadias, undescended testes, sacral sinus, delayed tooth eruption, spastic quadriplegia/tetraparesis and severe intellectual disability. MRI showed agenesis of corpus callosum(ACC) and mild dilatation of ventricular system. Other malformations found in this case include: cardiac defects (atrial septal defect (ASD)) and bilateral optic atrophy. Karyotype was 46,XY and MLPA confirmed a ~2 Mb deletion in WHS region.

CASE 2

The second case, a 4-year-old girl, is the only child of non-related parents. The pregnancy evolved with a threatening miscarriage and polyhydramnios. The child was born at term, by cesarean section (Apgar score 9, Wt = 2500 g, Ht = 48 cm, OFC = 31.5 cm). Postnatal development was delayed. Jacksonian seizures have been noted in the right half of the body. Physical examination revealed small size (Wt: −3.2 SD, Ht: −4.15 SD), microcephaly (OFC: −4.66 SD), dysmorphic face, congenital cataract, dental anomalies, sacral sinus, two tuberous hemangiomas, hypotonia. The patient also has ASD, ventricular septal defect (VSD) and patent ductus arteriosus (PDA). Karyotype was 46,XX and MLPA P096 Probemix confirmed a ~2 Mb deletion in WHS region.

CASE 3

**Figure 1:**
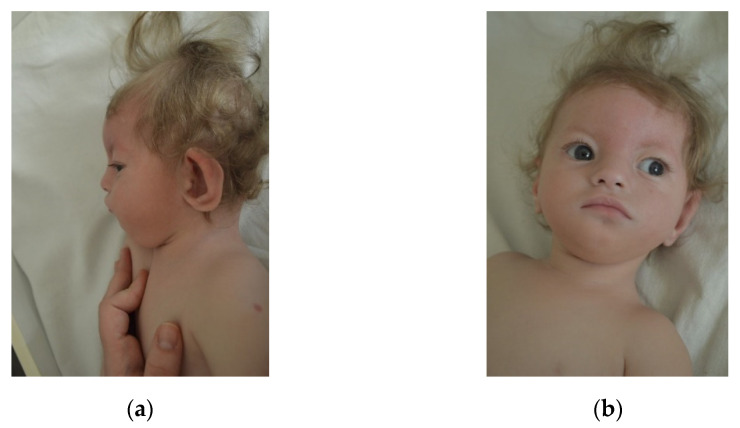
Craniofacial features of case 3. (**a**) lateral view (left side), (**b**) frontal view.

Case 3 is a 2-year-old boy, second child of an unrelated, apparently healthy couple, with no similar cases in the family. Pregnancy evolved with intrauterine growth retardation. The child was born at 36 weeks, by cesarean section (Wt = 1400 g, Ht = 30 cm, OFC = 28 cm). Postnatal development is delayed. The last evaluation (1 Y 6 Mo) revealed small size (Wt: −7.09 SD, Ht: −5.7SD), microcephaly (OFC: −4.81 SD), characteristic dysmorphic face (Figure 1).

**Figure 2:**
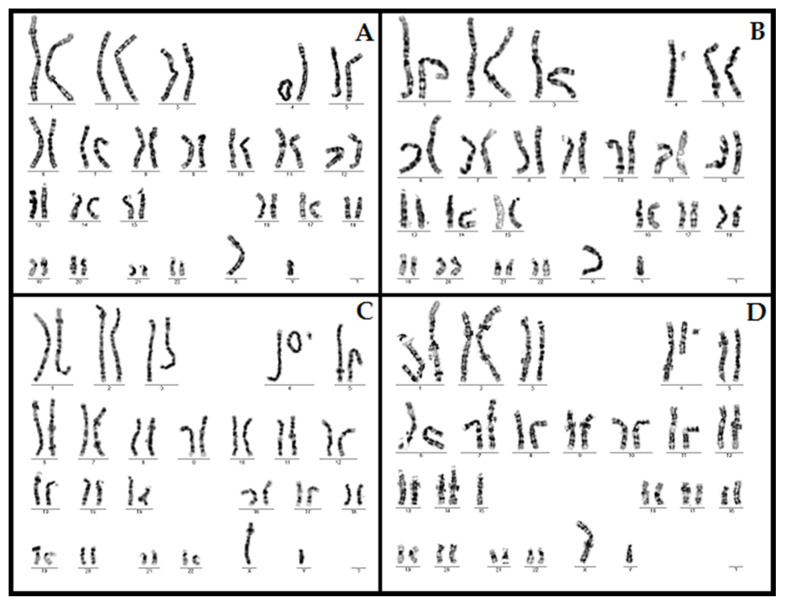
Karyotype in case 3: 46, XY,r(4)(p15.1q35)/46,XY,−4,+mar/47,XY,r(4)(p15.1q35),+mar/46,XY,der(4),+mar. (**A**) 46,XY,r(4)(p15.1q35); (**B**) 46,XY,−4,+mar; (**C**) 47,XY,r(4)(p15.1q35); (**D**) 46,XY,der(4),+mar.

**Figure 3:**
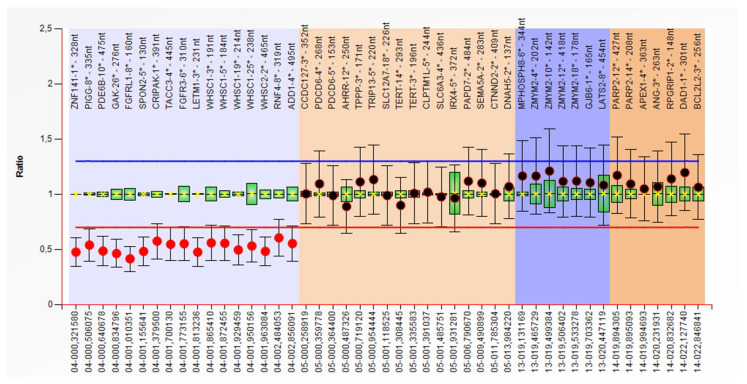
MLPA Follow-up kit P358: Heterozygous deletion in the terminal region of the 4p chromosome (2, 85 Mb) (case 3).

He also presents associated brachydactyly, bilateral transverse palmar crease, hypospadias, hypotonia and severe developmental delay. Imagistic investigations identified: ASD, pulmonary artery stenosis, hypoplastic corpus callosum. Karyotype was 46,XY,r(4)(p15.1q35)/46,XY,−4,+mar/47,XY,r(4)(p15.1q35),+mar/46,XY,der(4),+mar (Figure 2) and MLPA P358 Probemix confirmed a ~2.85 Mb deletion in WHS region (Figure 3 and P264 Probemix identified a deletion on 4q.

CASE 4

Case 4 is a 3-year-old male with severe developmental delay and multiple congenital anomalies. He is the first child of a young, unrelated couple. During the pregnancy (28 WA) an 8 mm cyst was identified in *cavum vergae*. At birth he presented respiratory distress (Apgar score 8, Wt = 2600 g, Ht = 49 cm, OFC = 33 cm). Postnatal development is delayed (cannot raise head, sit without help, or speak) and was characterized by seizures and recurrent respiratory infections. Last evaluation revealed small size (Wt: −5.61 SD, Ht: −3.78 SD), microcephaly (OFC: −2.75 SD), dysmorphic face, generalized muscle hypotonia, bilateral iris coloboma, and congenital cataract. Other anomalies associated: ASD, hypospadias, bilateral cryptorchidism/bilateral undescended testes and bilateral hydronephrosis. Brain MRI revealed postero-anterior interhemispheric cyst of the caudal portion of the *corpus callosum*. Karyotype was 46,XY and MLPA P036/P070 and P096 Probemix confirmed a ~2 Mb deletion in WHS region. Parental chromosomal analyses showed a paternal abnormal karyotype: 46,XY,1qh+,t(4;17)(p15.33;p13.3). No copy number variation was identified on 17p region by MLPA with P249 Probemix in the child.

CASE 5

**Figure 4:**
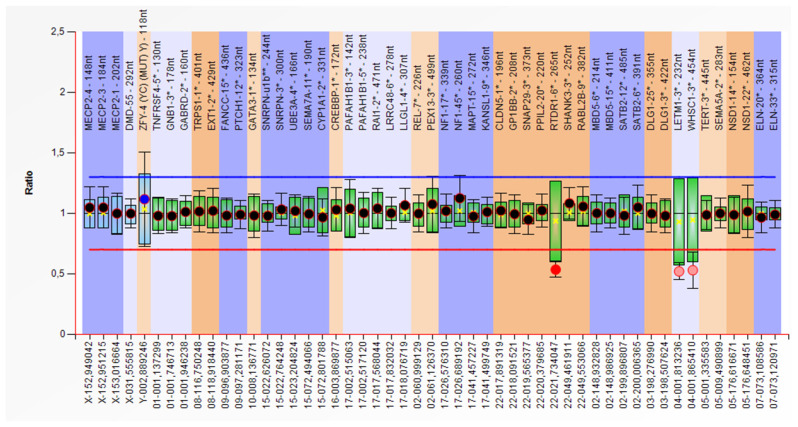
MLPA kit P245: Heterozygous deletion of the 4p16.3 chromosome and heterozygous deletion of 22q11.2 (*RTDR1* gene) (case 5).

Case 5 is a 1-year-old boy, the only child of an unrelated couple, with no other cases in the family. The pregnancy was uneventful, and the child was born at term (Apgar score 7, Wt-2700 g, Ht and OFC unknown). Postnatal evolution is severely delayed (can not raise head, does not speak). Last clinical examination revealed small size (Wt: −5.7 SD), microcephaly (OFC: −3.91 SD), dysmorphic face, and sacral sinus. The patient also has ASD, seizures, and frequent respiratory infections. Karyotype was 46,XY,del(4)(p16.3),del(22)(q11.23) and MLPA P245 Probemix identified a ~2 Mb deletion in WHS region in association with a small deletion in 22q11.2 region. A ~2 Mb 4p deletion was confirmed by P096 MLPA kit and the deletion in 22q11.2 in *RTDR1* gene was confirmed by P250 MLPA kit (Figure 4).

### 3.2. Large Deletions (>3 Mb)

CASE 6

Case 6 is a 5-year-old girl, the first child of an unrelated young couple. The family history is negative. She presented prenatal growth delay, and she was born prematurely (33 WA; Wt = 930 g, Ht = 38 cm, OFC = 36 cm, Apgar score 1). Postnatal development was delayed (raised head at 4 Mo, sat at 8 Mo). The last evaluation (4 Y 4 Mo) revealed: proportionate dwarfism (Wt: −2.6 SD, Ht: −4.08 SD), marked microcephaly (OFC: −7.41 SD), dysmorphic face, right iris coloboma, anodontia, left preauricular pit, muscle hypotonia, severe intellectual disability, and hearing loss. The patient also presents associated congenital heart defects (ASD, VSD, tricuspid insufficiency(TI), mitral and pulmonary insufficiency. Karyotype was 46,XX,del(4)(p16.1-pter) and a ~8 Mb deletion in WHS region confirmed by FISH.

CASE 7

**Figure 5:**
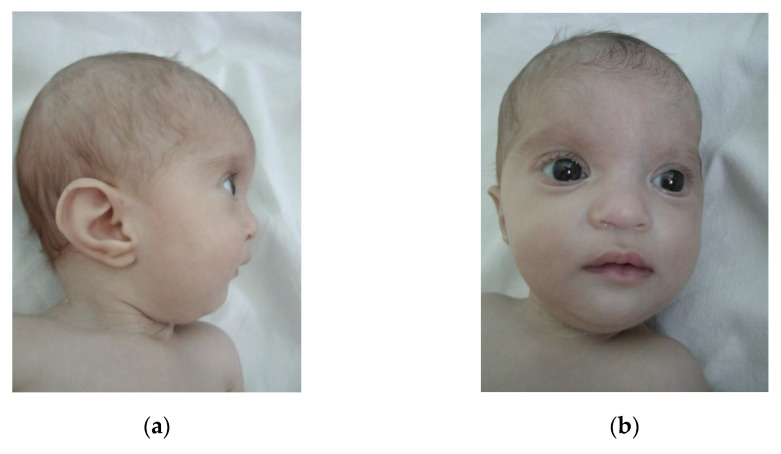
Craniofacial features of case 3. (**a**) lateral view(right side), (**b**) frontal view.

Case 7 is an 1-year-old girl, third child of a young, unrelated young couple, with no other cases in the family. Pregnancy was characterized by an imminent miscarriage (resolved by obstetrician) and an uneventful birth at term(Wt = 1600 g, Ht = 45 cm, OFC = 29 cm, Apgar score 9). The girl has a poor postnatal growth. Clinical evaluation (10 Mo) noted small size (Wt: −5.19 SD, Ht: −3.19 SD), microcephaly (OFC: −2.81 SD), characteristic dysmorphic face (Figure 5a,b), hypotonia, developmental delay, and hearing loss (bilateral stenosis of auditory canal). Echocardiography revealed: ASD and pulmonary stenosis; recurrent respiratory infections were also noticed. Karyotype was 46,XX,del(4)(p15.2-pter) and MLPA confirmed a ~22 Mb deletion in WHS region.

Clinical features of our patients are synthetized in Table 1.

## 4. Discussion

Wolf-Hirschhorn syndrome has a high clinical variability, but also a great variability of the deletion both in terms of size and etiological mechanism. Our selected cases illustrate this well. Most WHS patients had a “pure” deletion with no other cytogenetic anomalies (55%); the remaining had a more complex cytogenetic profile, such as derivative chromosome 4 resulting from an unbalanced translocation, ring 4 chromosome or a 4p-mosaicism [10].

Cases 6 and 7 had large deletions on the short arm of chromosome 4 that was observed on the karyotype. Patient’s 6 karyotype—46,XX,del(4)(p16.1-pter)—revealed an 8.2 Mb deletion. Thus, it falls in the category of average size deletions (5 to 18 Mb). The clinical features of this specific case are similar to the ones found in a case report of a much younger girl (8 months old), both reporting impairments of the auditory system, microcephaly, coloboma, congenital heart defects, and delayed development which are considered to be some of the diagnostic markers for WHS [11]. Case 7 is part of the third category of deletions in WHS, having a 22 Mb deletion–46,XX,del(4)(p15.2-pter). The phenotype of the two patients is more severe in terms of developmental delay, and cardiac impairment is also important. Both cases had hearing loss, aspect also illustrated by other studies in WHS cases with deletions greater than 5 MB [12,13]. In South et al.’s study, 13 of 32 patients have hearing loss, four of this has 4p deletions are smaller than 6 Mb, one pure deletion, and the other three show unbalanced translocations [14].

In cases 1, 2 and 4, the syndrome was caused by microdeletions (~2 Mb) that comprise the critical region of 4p16.3, and therefore cannot be identified on the karyotype. Following the cytogenetic evaluation of the parents, a balanced translocation between chromosomes 4 and 17 was found in the father of patient 4 (46,XY,1qh+,t(4;17)(p15.33;p13.3)). This was not surprising because approximately 15% of cases with WHS are generated by a mis-segregation of derivative chromosomes in meiosis of a carrier of balanced translocation [14]. The most common translocations in WHS are with the chromosome 8p, followed closely by translocations with 7p, 11p, 12p [15]. Patients with an unbalanced translocation usually present some deviation from classic clinical manifestations due to modification of the phenotype by the trisomy material [14]. In case 4, only the 2 Mb deletion in region 4p15.33→ter was important for the phenotype. However, the balanced translocation carried by the father of case 4 poses a problem in giving genetic counseling in future pregnancies. Thus, the parental couple had a 10–12% risk to have an abnormal embryo that associates either monosomy 4p15.33→ter and a small trisomy 17p13.3→ter either a small monosomy 17p13.3→ter and a trisomy 4p15.33→ter, in both situations is a high-risk pregnancy with birth of an abnormal child.

Case 3 analyses portrayed a complex chromosomal rearrangement: 46,XY,r(4)(p15.1q35)/46,XY,−4,+mar/47,XY,r(4)(p15.1q35),+mar. Using MLPA (kit P096, follow-up kit P264 and P358) only a 2.85 Mb size deletion was identified in the 4p16.3 region with no significant loss on the long arm of chromosome 4. We can presume in this case that a mechanism characterized by a telomere-to-telomere fusion which generates a pseudo-complete ring chromosome, associated with a small loss of genetic material is responsible for microdeletion in terminal part of short arm of chromosome 4. However, such cases are very rare and represent not more than >1% of WHS [15,16].

In case 5, MLPA test confirmed the presence of two microdeletions: one 4p16.3 and other 22q11.23. The first microdeletion was pathogenic and produced WHS. The second microdeletion involved the absence of the *RTDR1* gene. The function of the protein encoded by this gene is yet unknown, but no pathogenicity has been associated with mutation in the *RTDR1* gene. In order to characterize the 22q11.23 anomaly and establish the pathogenicity, it is necessary to evaluate the parents as well as complete the investigations with CMA to exclude a microdeletion that includes the *RTDR1* gene (between genomic positions 20.652996-21.795392, which border the anomaly identified by MLPA P250 Probemix).

Between 90% and 100% of reported cases of WHS present epilepsy [3], which is usually manifested in the first 3 years of life with a peak in incidence between 6 and 13 months. Fever triggered the seizure in infancy in about 70% of the 87 cases presented by Battaglia et al. [17]. In a study of 83 patients Bi et al. proposes that the manifestations and intensity of seizures is attributed to the synergistic effect of several genes, such as *PIGG*(phosphatidylinositol glycan anchor biosynthesis class G), *CPLX1*(complexin-1) and *LETM1*(leucine zipper and EF-hand containing transmembrane protein 1) in the WHS region; genes located within this 0.3 Mb defined as a seizure susceptibility interval (between 0.6 and 0.9 Mb from the terminus) [12]. All of our cases cover this area of susceptibility, and the absence of seizure may be due to the young age of some patients (cases 3 and 5). Alternatively, seizures could have been very mild, and they were not noticed by caregivers.

Intrauterine growth restriction is a clinical sign present in most cases of WHS (80–90% cases) [3,18]. In our study, IUGR was detected in a relatively small number of cases (with deletions larger than 2 MB) but postnatal failure to thrive was a constant feature presented in all reported patients.

Heart defects are often mild in WHS, with the most common heart defect being an atrial septal defect, followed by pulmonary valve stenosis, tetralogy of Fallot, ventricular septal defect and patent ductus arteriosus (PDA). Structural heart defects occur in approximately 50% of cases [3,17]. Catela et al. (2009) proposed in their study the deletion of *FGFRL1*(fibroblast growth factor receptor like 1) gene as a plausible candidate for the occurrence of cardiac anomalies [13]. In our study, MLPA analysis revealed haploinsufficiency of *FGFRL1* gene in all seven cases, all but one presented mild cardiac defect. In case 6 the cardiac anomaly was more complex; the added severity can be attributed to the larger size of the chromosomal deletion.

In the case of WHS, as a contiguous gene syndrome, it is considered that the smaller the deletion is the less severe are the clinical manifestations [7]. In our cohort study we did not identify major differences between microdeletions and large deletions. This might be explained by the existence of a critical region of the syndrome that determines most of its features [19]. Thus, *WHSC1*(Wolf-Hirschhorn syndrome candidate 1) gene, whose heterozygous deletions were observed in all seven cases, could be considered responsible for developmental delays, facial dysmorphisms, and short stature [20]. Interestingly, in two cases presented by Zollino and her team [21,22], they have reported that their cases of microdeletions smaller than 2.8 Mb or none at all only showed a mild phenotype with malformations usually absent and a normal head circumference. However, in the cases 1, 2, and 4 presented by us, we have noted dysmorphic face, cardiac defects, multiple congenital anomalies and severe postnatal development which hints towards the involvement of genes other than the ones in the critical region. However, we have identified oral issues alongside psychomotor delays in case 1, 2, and 6(anodontia) similar to the findings of Limeres et al. [23].

Concerning genetic investigations, in our experience, combined MLPA (P036/P070 or P245 followed by P279, P358, P096 or another follow up kit for 4p region) use is a reliable and rapid screening tool. The advantage of MLPA follow up kit is that they contain multiple probes for genes located in the region and in limited number of cases. Such a kit could be sufficient to establish deletion size and genes involved and this may represent an advantage compared to classical FISH. This represents an alternative strategy for cases when CMA is not easily accessible, but for the rest, CMA remains the gold standard technique for WHS diagnosis.

Even if our study did not manage to identify major differences between small size deletions and large deletions, one possible explanation for that could be reduced number of participants. Despite this, we consider that MLPA is a reliable tool for the diagnosis of WHS and, considering the cheaper and more easily accessible nature of this test, MLPA could be used as a first-tier test in the diagnosis of WHS as it is presented in this study.

## 5. Conclusions

Wolf-Hirschhorn syndrome is a rare genetic disorder characterized by complex clinical manifestations and a severe prognosis. Although the general clinical aspects are frequently suggestive for the syndrome, the wide and varied spectrum of manifestations may cause difficulties in diagnosis. Our cases illustrate the high variability of WHS: facial dysmorphism and developmental delay are common, but renal and brain defects may be found and should be looked for; hearing loss is also associated with large deletions. Therefore, cytogenetic, and molecular tests are imperative in these patients. Despite the fact that 85% of cases are de novo mutations, in 15% of cases the derivative chromosomes generated by a meiotic mis-segregation of a balanced translocation present in one of the parents were identified. Hence, different genetic investigations(CMA, MLPA, FISH, karyotype) are requested in order to complete the diagnosis in patients with WHS, as well as the chromosomal analysis of the parents becoming mandatory for assessing the risk of occurrence of another case in family. Although CMA is the gold standard test for WHS diagnosis, depending on the possibilities of the laboratory, MLPA could be used as a screening method before performing CMA in specific cases.

## Figures and Tables

**Table 1:** Clinical characteristic of the WHS patients. OFC—occipital-frontal circumference, ASD—atrial septal defect, VSD—ventricular septal defect, PDA—patent ductus arteriosus, IUGR—Intrauterine growth restriction.

Name	Case 1	Case 2	Case 3	Case 4	Case 5	Case 6	Case 7
Age (years)	13	4	2	3	1	5	1
Sex	M	F	M	M	M	F	F
Uneventful pregnancy	+	−	−	−	+	−	−
Weight at birth (g)	3600	2500	1400	2600	2700	930	1600
Weight last exam (SD)	−0.89	−3.2	−7.09	−5.61	−5.75	−2.6	−5.19
Height at birth (cm)	52	48	30	49	-	38	45
Height last exam (SD)	−2.39	−4.15	−5.75	−3.78	-	−4.08	−3.19
OFC at birth (cm)	36.5	31.5	28	33	-	36	29
OFC last exam (SD	−1.23	−4.66	−4.81	−2.75	−3.91	−7.41	−2.81
Prenatal growth delay	−	−	+	+	−	+	−
Postnatal development delay	+	+	+	+	+	+	+
Born at term	+	+	+	+	+	−	+
IUGR	−	+/−	+	−	n.a	+	+
Karyotype	46,XY	46,XX	46,XY,r(4)(p15.1q35)/46,XY,−4,+mar/47,XY,r(4)(p15.1q35),+mar/46,XY,der(4),+mar/46,XY	46,XY	46,XY,del(4)(p16.3),del(22)(q11.23)	46,XX,del(4)(p16.1-pter)	46,XX,del(4)(p15.2-pter)
Size of deletion	~2 MB	~2 MB	~2.85 MB	~2 MB	~2 MB	~8 MB	~22 MB
Dysmorphic face	+	+	+	atypical	+/−	+	+
Microcephaly	−	+	+	+/−	+/−	+++	+/−
Dental anomalies	+	+	−	−	−	+	−
Delayed tooth eruption	+	+					
Anodontia						+	
Ear anomalies	+	+	+	+	−	+	+
Left preauricular pit						+	
Hearing loss	−	−	−	−	−	+	+
Ocular defects	+	+	−	+	−	+	−
Defect of lacrimal system	+						
Congenital cataract		+		+			
Tuberous hemangiomas		+					
Bilateral optic atrophy	+						
Iris coloboma				+		+	
Intellectual disability	Severe	Mild	Severe	Severe	n.a.	Severe	n.a.
Brain anomalies	+	−	+	+	−	−	−
Spastic quadriplegia	+						
Hypoplastic corpus callosum	+		+				
Corpus callosum cyst				+			
Seizures	−	+	−	+	+	−	−
Jacksonian seizures		+					
Immunodeficiency	+	−	−	+	+	−	+
Cardiac defects	+	+	+	+	+	+	+
ASD	+	+	+	+	+	+	+
VSD		+				+	
PDA		+					
Tricuspid insufficiency						+	
Mitral insufficiency						+	
Renal anomalies	−	−	−	+	+	−	−
Pulmonary defects	+		+	+	+	+	+
Pulmonary insufficiency						+	
Pulmonary stenosis			+				+
Infections	+			+	+		+
Hypotonia		+	+	+		+	+
Diastasis recti, hypospadias, undescended testes	+		+	+			

## Data Availability

All the data are available in the present paper.
